# Automating Endoscope Motion in Robotic Surgery: A Usability Study on da Vinci-Assisted *Ex Vivo* Neobladder Reconstruction

**DOI:** 10.3389/frobt.2021.707704

**Published:** 2021-11-25

**Authors:** Tommaso Da Col, Guido Caccianiga, Michele Catellani, Andrea Mariani, Matteo Ferro, Giovanni Cordima, Elena De Momi, Giancarlo Ferrigno, Ottavio de Cobelli

**Affiliations:** ^1^ Neuro-Engineering and Medical Robotics Laboratory (NEARLab), Department of Electronics, Information and Bioengineering, Politecnico di Milano, Milan, Italy; ^2^ Haptic Intelligence Department, Max-Planck-Institute for Intelligent Systems, Stuttgart, Germany; ^3^ Division of Urology, European Institute of Oncology, IRCCS, Milan, Italy; ^4^ Excellence in Robotics and AI Department, Sant’Anna School of Advanced Studies, Pisa, Italy

**Keywords:** robotic surgery, da Vinci surgery, surgery automation, endoscope automation, oncology, urology, neobladder reconstruction

## Abstract

Robots for minimally invasive surgery introduce many advantages, but still require the surgeon to alternatively control the surgical instruments and the endoscope. This work aims at providing autonomous navigation of the endoscope during a surgical procedure. The autonomous endoscope motion was based on kinematic tracking of the surgical instruments and integrated with the da Vinci Research Kit. A preclinical usability study was conducted by 10 urologists. They carried out an *ex vivo* orthotopic neobladder reconstruction twice, using both traditional and autonomous endoscope control. The usability of the system was tested by asking participants to fill standard system usability scales. Moreover, the effectiveness of the method was assessed by analyzing the total procedure time and the time spent with the instruments out of the field of view. The average system usability score overcame the threshold usually identified as the limit to assess good usability (average score = 73.25 > 68). The average total procedure time with the autonomous endoscope navigation was comparable with the classic control (*p* = 0.85 > 0.05), yet it significantly reduced the time out of the field of view (*p* = 0.022 < 0.05). Based on our findings, the autonomous endoscope improves the usability of the surgical system, and it has the potential to be an additional and customizable tool for the surgeon that can always take control of the endoscope or leave it to move autonomously.

## Introduction

While new generations of robot-assisted surgical systems approach the market (da Vinci®, Senhance®, Versius®, and Revo-i®), robot autonomy is still away from clinical application (Connor et al., 2020; Kranzfelder et al., 2013). Modern robotic systems still require the operator to manually control at least two surgical tools and an endoscope (Attanasio et al., 2021). This approach requires frequent use of foot pedals to change the viewpoint, freezing the teleoperation of the surgical instruments for adjusting the workspace, or switching between instruments. This often leads to disrupted operational workflows, extended overall procedural times, and task overload (Zheng et al., 2010).

Many current research lines are oriented toward increasing the level of robot autonomy to assist surgeons and to help in reducing their workload, especially for trivial but time-consuming tasks. Pandya et al. identified endoscope control as a near-term development for automation in surgical robotics (Eslamian et al., 2020; Pandya et al., 2014). In a general sense, autonomous endoscope systems take care of optimal viewpoint without requiring continuous readjustments from the operator.

Related works show few robotic endoscope manipulators being integrated with autonomous control strategies. The Automated Endoscopic System for Optimal Positioning (AESOP^®^, Computer Motion Inc., Santa Barbara, CA, USA) (Jacobs et al., 1997) autonomously centered the user point of gaze using an infrared (IR) eye-tracking system (Ali et al., 2008). The AutoLap^®^ was integrated with a modality to follow a tool designated by the user (Wijsman et al., 2018). In some previous experiments, we investigated the use of an autonomous endoscope navigation system referred to as SCAN (System for Camera Autonomous Navigation) (Mariani et al., 2020; Da Col et al., 2020).

To the best of our knowledge, no intraoperative or *ex vivo* realistic experiments have been carried out on this topic yet. Animal *ex vivo* models have been extensively tested and validated through the years for the simulation of various urological procedures (Al-Jabir et al., 2020). Several urological procedures have been effectively simulated deploying different animal *ex vivo* anatomical parts. Urethrovesical anastomosis has been obtained by coupling chicken gizzard (simulating the resected bladder neck) with tracts of either the esophagus or rectum (Sotelo et al., 2009; Laguna et al., 2006). Inverted-U configuration neobladder reconstruction was reproduced using pig intestine tracts (Singh et al., 2021).

We foresaw the potential success of autonomous endoscope navigation in all those procedures that may require a wide workspace and, consequently, an extensive and frequent manipulation of the view field. This consideration led to the decision of setting up our investigation focusing on orthotopic neobladder reconstruction. We selected and standardized a subset of procedural steps (see *Ex vivo experimental setup* section) of the “Shell” technique (Bianchi et al., 2020) in which endoscope manipulation might be further optimized as well as autonomy may provide fruitful assistance.

Our present contribution is therefore a preclinical validation of the SCAN. A usability study, involving surgical residents and surgeons with different levels of expertise performing orthotopic neobladder reconstruction on an *ex vivo* porcine model, was carried out. We aim at demonstrating the system usability for surgeons in a realistic urological scenario and the potentials of autonomous endoscope navigation in optimizing the operational workflow in robotic surgery.

## Materials and methods

### Outline of the surgical procedure

Orthotopic neobladder has risen during the last decades as an alternative to the ileal conduit (Almassi and Bochner, 2020) (incontinent diversion to skin with external synthetic reservoir) and the Indiana pouch (Chennamsetty and Chan, 2018) (continent diversion to skin with internal biological reservoir). This procedure ensures clear advantages in terms of quality of life for the patient such as partial or complete continence, in case the natural urethral sphincter is preserved, and no external anatomical/aesthetic changes due to diversion to the abdominal wall. The absence of diversion and any form of external reservoir makes this approach more appealing to youth patients allowing for a more active and breezy lifestyle (Spencer et al., 2018; Mizzi, 2020).

Even though this surgical procedure has been proposed in many variants throughout the years, a comprehensive outline can be summarized by the following steps:

i) Bowel segment selection, distension, and measurement.
ii) Segment separation through stapling.
iii) Lateral ileal–ileal anastomosis.
iv) Anastomosis between the urethra and the middle section of the segment.
v) Detubularization of the segment.
vi) Neobladder plication and suturing.
vii) Ureteroileal anastomosis.

A video representation of the full “Shell” technique we aimed at reproducing can be found at Bianchi et al. (2020).

### *Ex vivo* experimental setup

**FIGURE 1:**
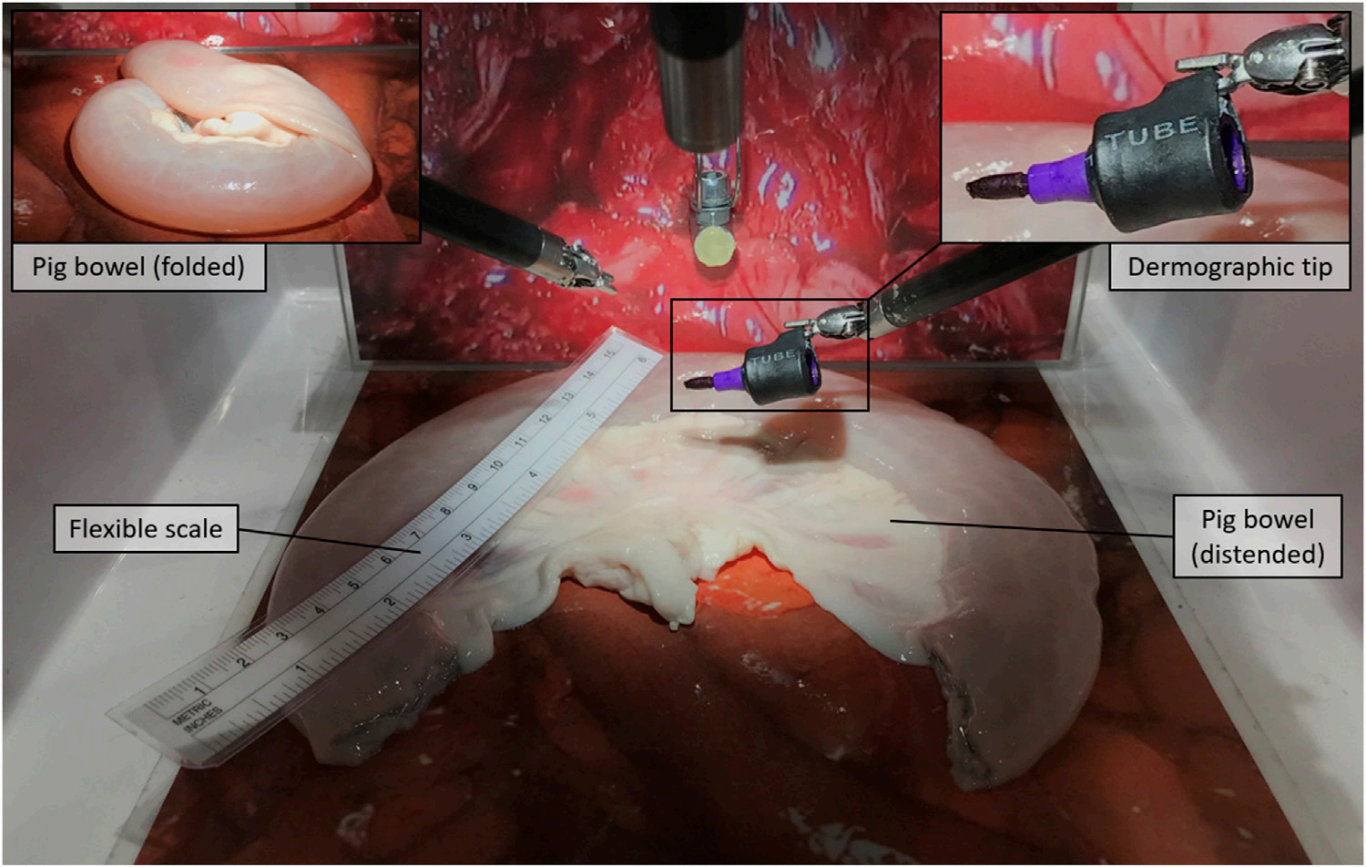
Distension and measurement, Step (i): The pig bowel is presented in a folded configuration to be distended and measured by means of a flexible scale. The figure also shows the dermographic tip that is later used in step (v).

For the experiment, we selected three of the neobladder reconstruction steps (i.e., i, v, and vi) to be reproduced by using small tracts of pig bowels. The bowel segment selection step (usually around 40 cm saving the last 15 cm before the ileocecal valve) was removed from (i) to reduce the amount of animal tissue wasted during each experiment and the potential variability in the resulting neobladder dimensions due to incorrect measurements. The same logistical and ethical concerns lead to the decision of removing the segment separation (ii) and the ileal–ileal anastomosis (iii) steps to avoid unnecessary use of disposable stapler cartridges. Steps (iv) and (vii) were removed because they would have involved other organs farther than the bowel. As a result, the surgeon was provided directly with a standardized, precut, 30-cm-long pig bowel tract that was mechanically presealed at the two extremities and partially filled with saline solution to simulate the semifull bowel consistency. The presealed pig bowel sample was therefore presented lying in a folded configuration to simulate a random configuration inside the peritoneal cavity (Figure 1).

**FIGURE 2:**
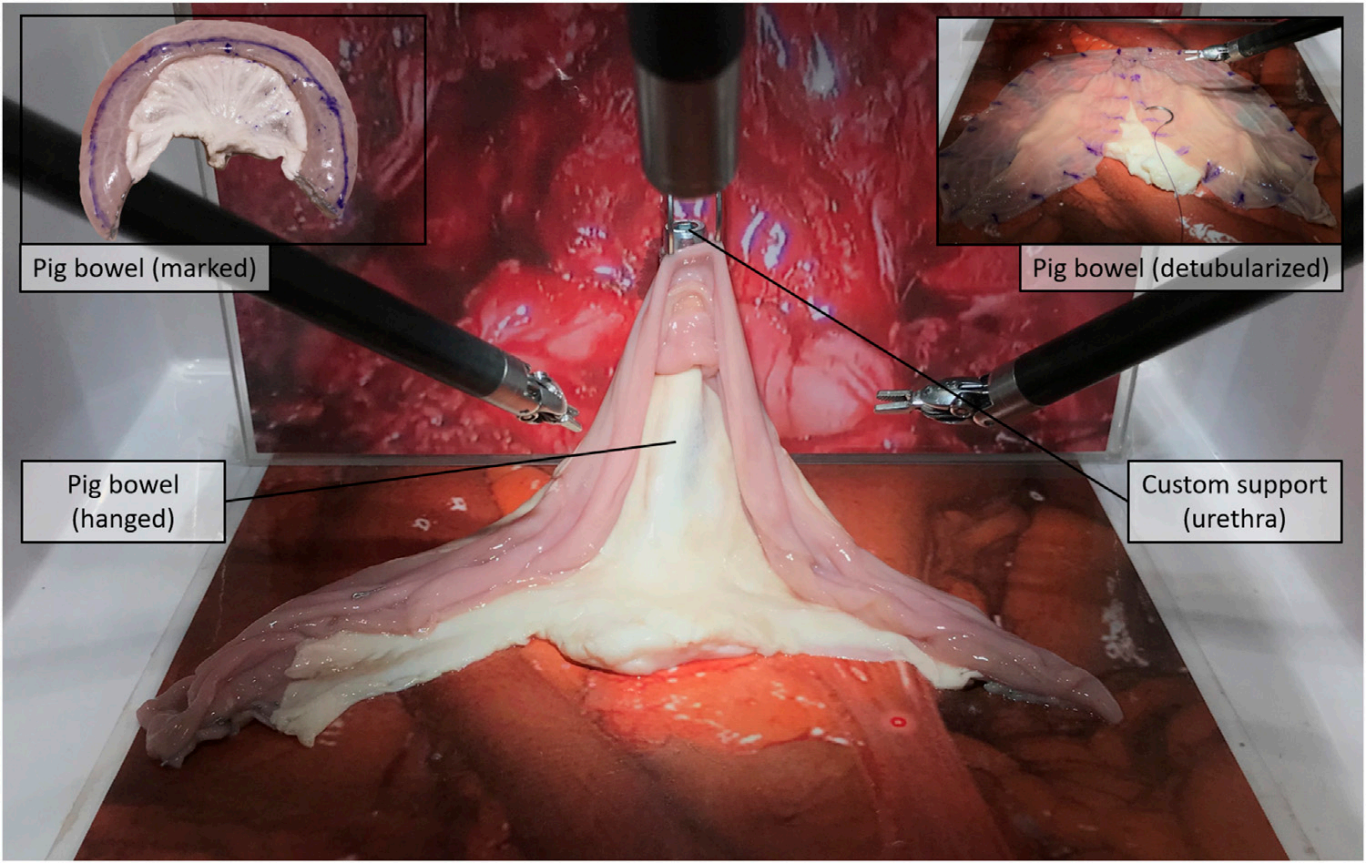
Detubularization, step (v): Surgeons mark the sample with the dermographic tip along the detubularization line, while the technical assistant then cuts along that marked line with a scalped.

**FIGURE 3:**
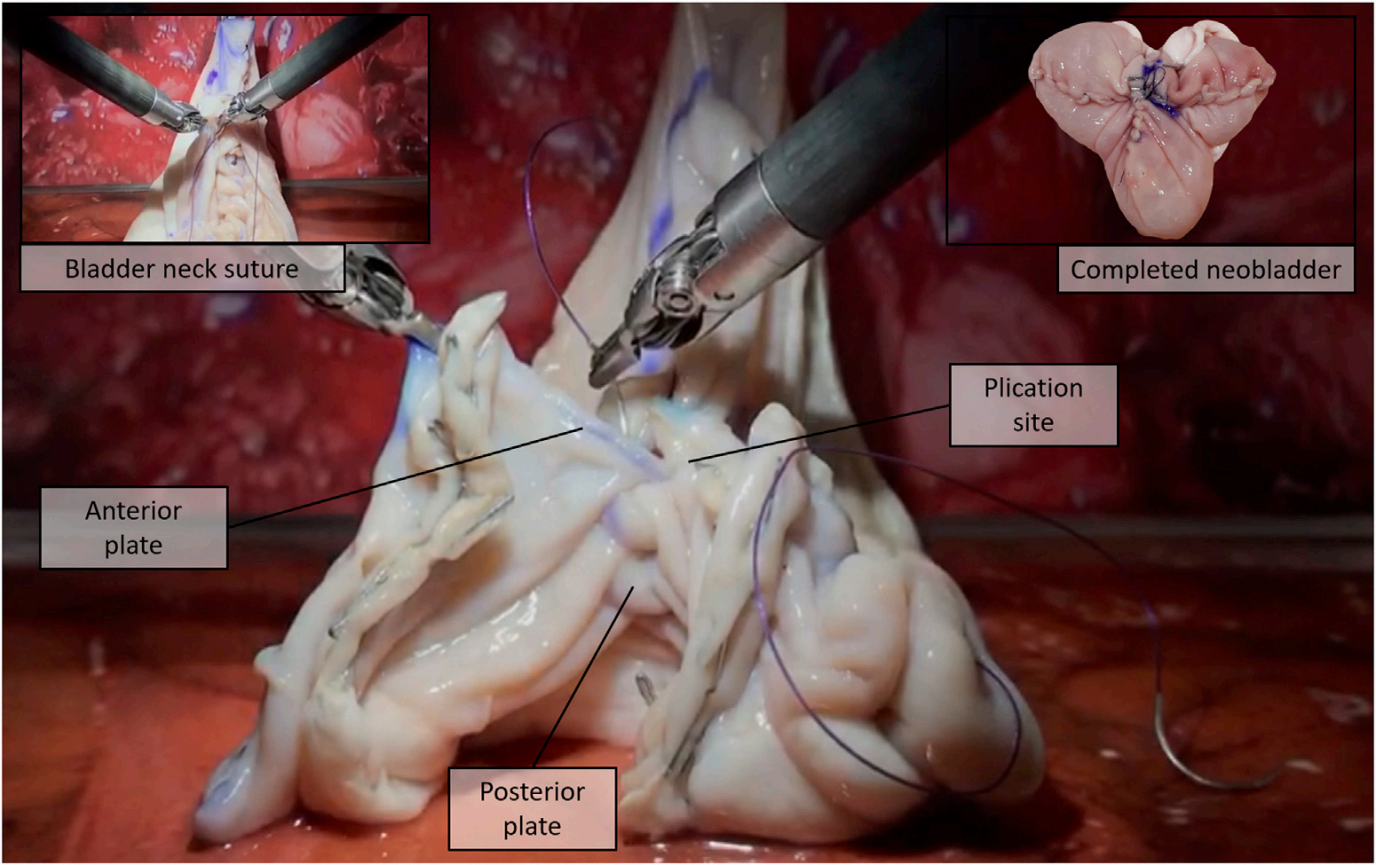
Plication and suturing, step (vi): The detubularized bowel is sutured to obtain the final “Shell” neobladder [16].

i) Distension and measurement—Surgeons were asked, as a first step, to manipulate the bowel until maximum distension was reached, and to measure its total length with a standard surgical flexible scale (Figure 1); participants were not informed of the real length of the samples nor of their standardized nature. This was mostly done to keep them motivated during the measurement phase.
v) Detubularization—Since electrocautery was not available at the testing facility, participants were asked to mark the distended bowel sample with a dermographic pen moving along the same detubularization path they would have followed with the actual electrocautery. A special custom-made support was realized to allow the da Vinci Large Needle Driver® to firmly hold a dermographic tip (Figure 1). Immediately after, the sample was temporarily removed from the operating space, manually detubularized by a surgical assistant with a scalpel, and repositioned in the endoscopic field of view. The sample was then attached to a custom-made hook support and positioned as it had already been anastomized to the urethra (Figure 2).
vi) Plication and suturing—Finally, two 15-cm-long Monocryl® 3–0 UR-6 sutures were used to perform a standardized neobladder plication and suturing with eight passages for the posterior plate, three for the neobladder neck, and five for the anterior plate performing a simplified shell-type reconstruction (Figure 3).

A video showing the entire procedure carried out by the surgeons during the experiment can be found in the Supplementary Material.

### System architecture

**FIGURE 4:**
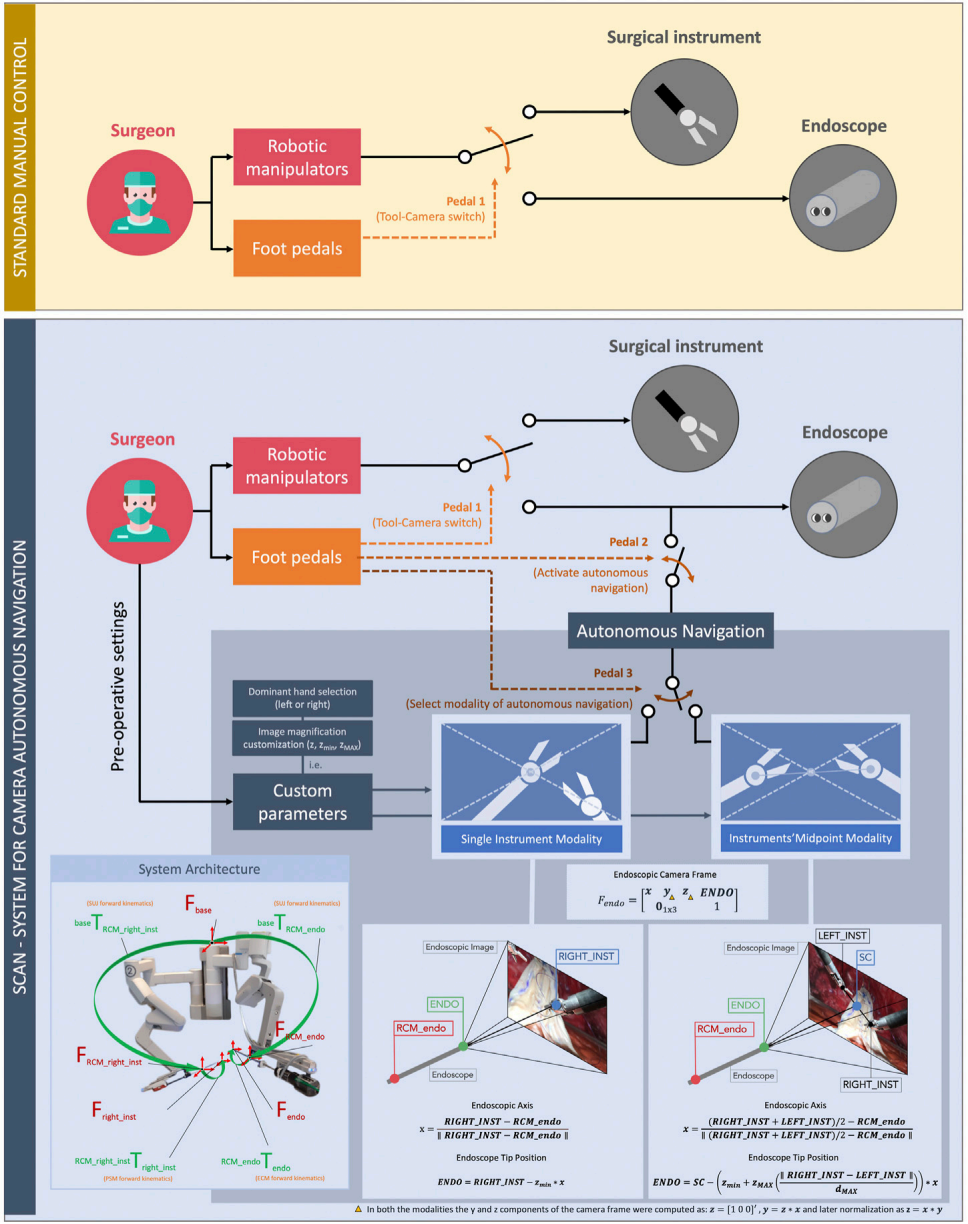
Comparison between the standard manual control (up, yellow) and the autonomous control (down, blue) of the endoscopic camera. In the conventional framework of manual control of the endoscope, the surgeon switches between using the robotic manipulators for controlling the surgical instruments and the endoscope by means of a pedal (pedal 1). In our new framework, the surgeon can activate the autonomous navigation of the endoscope whenever needed (pedal 2). Foot-pedal control also allows selecting the autonomous navigation submodality (pedal 3). These are the *single instrument modality* and the *midpoint modality of the instruments*. On the bottom of the figure, the mathematical formulation of these modalities, together with a supporting image of the overall system architecture used in this study, are shown.

The da Vinci Research Kit was chosen to carry out this study. The da Vinci Research Kit is an open-source mechatronic platform (Kazanzides et al., 2014) featuring customized control electronics, firmware, and software, integrated with hardware components of the first generation of the da Vinci surgical system^®^ (sometimes called classic). The platform is composed of a patient side, featuring mainly the robotic arms mounting the surgical instruments, and a surgeon console integrated with interfaces for controlling the patient side. In particular, the patient side is composed of four robotic arms: up to three patient side manipulators (PSMs), mounting the interchangeable surgical instruments used to perform different procedures on the patients, and the endoscopic camera manipulator (ECM), holding the full HD stereo endoscope. All the robotic arms composing the patient side are linked together through a closed kinematic chain composed of the setup joints (SUJs), a series of links and passive joints that allow registration through direct kinematics of all the robotic arms with respect to the same reference frame (i.e., F_base_, the robot base frame; all the system frames and transformation are shown in Figure 4). The surgeon side consists of two robotic arms called master tool manipulators (MTMs), used by the surgeons to teleoperate the PSMs and, normally, the ECM. The surgeon console is also integrated with high-resolution stereo-viewer (HRSV) for immersive stereovision and pedals with different functions (e.g., switching from surgical instruments to endoscope control and freezing teleoperation for hand repositioning due to workspace constraints). Two PSMs mounting Large Needle Driver® or Cadiere Forceps®, depending on the surgeon's choice, and a straight 12-mm endoscope for vision was used for the experiments.

### Autonomous endoscope navigation

We previously introduced the SCAN (System for Camera Autonomous Navigation) both in a virtual reality training scenario (Mariani et al., 2020) and in dry-lab using the da Vinci Research Kit (Da Col et al., 2020). The algorithm controls autonomously the position of the camera (tip of the endoscope, **ENDO** in Figure 4) based on the surgical instruments position. The present work consists of a preclinical usability study. It involves the possibility of tailoring the system features based on the user preferences (i.e., dominant hand selection, endoscope position offset adjustment, and image magnification customization). It includes wet-lab testing in an experimental scenario closer to the real surgical application, involving a population of trained urological surgeons.

The key design principles of the SCAN were to guarantee an adequate viewpoint throughout the procedure, as well as good usability and intuitiveness of the system. In general, the system can continuously compute the surgical instrument poses and move the endoscope accordingly, resulting in an ongoing adjustment of the field of view. Intraoperatively, the surgeon is able to switch between two different navigation modalities by pressing a foot pedal on the surgeon console, as can be seen in Figure 4.

i) Single instrument modality—The field of view is constantly maintained centered on the right surgical instrument (or the left one, depending on the preference of the surgeon), and the distance between the endoscope tip and the instrument is maintained constant, resulting in a consistent magnification of the scene. Fixed parameters such as the lateral offset of the tool with respect to the center of the field of view and the amount of magnification can be preoperatively customized depending on the preference of the surgeon.

This modality was modeled by the following equations:

$$x=\frac{\mathrm{RIGHT}\_\mathrm{INST}-\mathrm{RCM}\_\mathrm{endo}}{\left\|\mathrm{RIGHT}\_\mathrm{INST}-\mathrm{RCM}\_\mathrm{endo}\right\|}$$

$$\mathrm{ENDO}=\mathrm{RIGHT}\_\mathrm{INST}-z_{\min}x$$

In the equations, the vectors are in bold, and they are all expressed with respect to the same reference frame (F_base_). **x** is the endoscope newly computed focal axis, obtained from **RIGHT_INST**, i.e., the right surgical instrument end-effector position (or left, depending on the dominant hand of the surgeon) and **RCM_endo**, the position of the endoscope remote center of motion. **ENDO** is the newly computed desired endoscope tip position, and z_min_ is the zooming factor, customizable by the user. For this experiment, the value of z was 0.05 m < z_min_ < 0.15 m (fixed for each surgeon). Note that the newly computed endoscope position always belongs to the segment connecting the instrument end-effector and the endoscope remote center of motion. All the terms composing the equations can be found in Figure 4.

ii) Midpoint modality of the intruments—The field of view is centered on the midpoint of the two surgical instruments, and the magnification of the scene is dependent on the relative distance between the m. This is for maintaining both the instruments inside the field of view of the surgeon and for providing the surgeon with the possibility to modify the image magnification intraoperatively. When the tools are closer to each other, the endoscope moves closer to them (thus, providing a close-up view). When the tools are far away from each other, the endoscope moves in the opposite direction, resulting in a wide view over the scene (Figure 4). Minimum and maximum magnification can be customized pre-operatively depending on the preference of the surgeon.

This modality was obtained by the following equations:

$$\mathrm{SC}=\frac{\mathrm{RIGHT}\_\mathrm{INST}+\mathrm{LEFT}\_\mathrm{INST}}{2}$$

$$x=\frac{\mathrm{SC}-\mathrm{RCM}\_\mathrm{endo}}{\left\|\mathrm{SC}-\mathrm{RCM}\_\mathrm{endo}\right\|}$$

$$\mathrm{ENDO}=\mathrm{SC}-\left(z_{\min}+z_{\mathrm{MAX}}\left(\frac{\left\|\mathrm{RIGHT}\_\mathrm{INST}-\mathrm{LEFT}\_\mathrm{INST}\right\|}{d_{\mathrm{MAX}}}\right)\right)x$$

Once again, in the above equations, the vectors are in bold, and they are all expressed with respect to F_base_. The **SC** (scene center, the element resulting at the center of the endoscope images) was computed from **RIGT_INST** and **LEFT_INST**, the positions of the right and the left surgical instruments, respectively. **ENDO**, the endoscope desired position, was a function of z_min_ = 0.1m, z_MAX_ = 0.1 m, and d_MAX_ = 0.2m, which are the minimum/maximum zoom allowed and the maximum distance between the tools, respectively. These values were set to match the task workspace, but preoperatively, the surgeon had the possibility to customize them. **x** again is the camera new focal axis. Even in this case, the endoscope position always belongs to the segment connecting the **SC** and the remote center of motion. All the terms composing the equations can be found in Figure 4.

In both the modalities, the y and z components of the endoscope frame (F_endo_) were computed as z = [1 0 0]′, y = z × x, and later normalized as z = x × y.

Furthermore, the surgeon can always interrupt the autonomous endoscope adjustment by pressing a pedal and, if required, directly manipulate the endoscope (as currently done in commercial da Vinci® systems). The desired autonomous navigation modality can be reactivated by means of another pedal.

### Acquisition protocol

**FIGURE 5:**
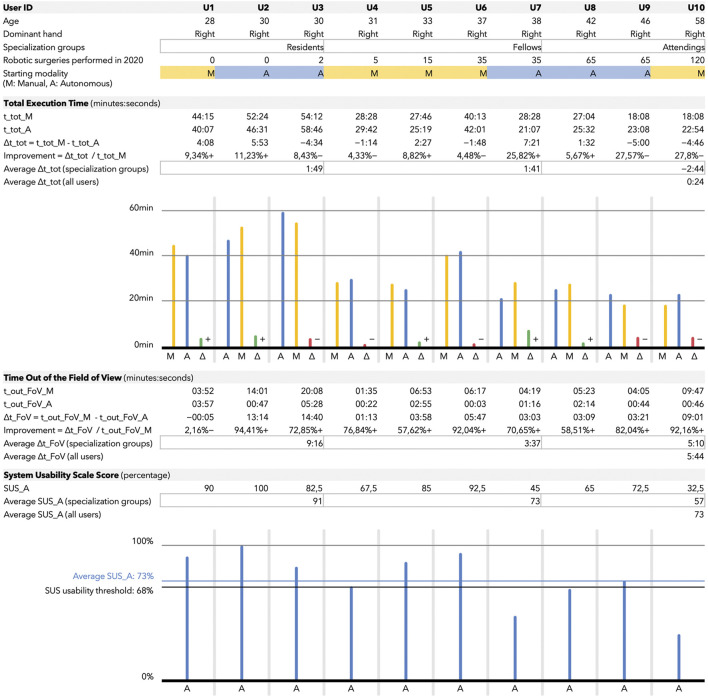
The figure is composed of four blocks containing tables and histograms. Users are ordered, from left to right, with increasing expertise. The expertise is determined by the number of robotic surgeries they performed in 2020 and their belonging to one of the following three groups: residents, fellows, and attendings. These groups, referred to as specialization groups in the table, are also used to compute group-averaged metrics. The first block contains user-related information, i.e., age, dominant hand, specialization groups, expertise, and starting modality. The starting modality (randomized among all the users) could be either M (yellow) for manual endoscope control and A (blue) for autonomous. The second block contains the total execution time (t_tot)-related metrics, expressed in minutes:seconds if not specified differently. A delta is computed as time in manual minus time in autonomous mode. For this reason positive values correspond to a total execution time reduction for the users when assisted by the autonomous system. Also, a variable called improvement is computed, which corresponds to the time (with respect to the total execution time in manual) gained or lost by the user when they exploited the autonomous endoscope navigation. Once again, the plus near the percentage symbol corresponds to a gain in time with the autonomous modality. The third block contains the data related to the time out the field of view (t_out_FoV). The considerations made for the total execution time table remain valid also for this one. The fourth block refers to the System Usability Scale score (SUS_A) used to assess the usability of the autonomous modality only. All the values in the table are expressed as percentage. Data for each user are also reported in the histogram. The blue horizontal line (at 73%) represents the average score among all the users; the one at 68% is the threshold usually considered to assess a good usability of a system.

The user study involved 12 medical doctors (see Figure 5) affiliated with the European Institute of Oncology (Milan, Italy), four of which are residents in urology and eight are specialized urologists. All of them had previous experience with teleoperated robots. Only 10 participants successfully finished the acquisition session, and the two incomplete trials (surgeons had to interrupt the session due to external factors) were discarded. As a first step, surgeons were asked to complete a pre-experiment survey to assess their clinical experience (reported in the upper part of Figure 5). Right after, they were introduced to the functioning of the autonomous navigation and left to familiarize with the system for 5 min.

Considering, in general, the high variability in style between surgeons, and specifically the diverse level of expertise in our sampled population, we decided to have each surgeon perform the neobladder reconstruction twice, once using manual and once using autonomous endoscope control. This redundant acquisition allowed us to evaluate the influence of the autonomous control modality (versus manual) at the individual level and, therefore, independently from the level of expertise of the surgeon. The order in which users performed the task undergoing manual or autonomous endoscope navigation was randomized with permuted block randomization. This adjustment was intended to compensate for the potential learning and fatigue effects connected to repeating the same procedure over a short period of time. Participants were explicitly requested to interrupt the autonomous navigation only if the SCAN was providing a suboptimal viewpoint.

One of the technical investigators played as assistant to constantly monitor the operating field interacting with the surgeon to timely provide the required external tools (such as dermographic tip, flexible scale, sutures, etc.) or adjustments (catheter repositioning, object grasping, etc.). Finally, a post-experiment survey (see the *System usability scale* section) was completed to derive a qualitative assessment and usability estimate of the system.

The user study took place at the Leonardo Robotics Laboratory of Politecnico di Milano in September 2020. All the *ex vivo* samples came from traceable food-grade butchered animals and were safely stored. The process was audited and approved by the ethical committee and the safety regulation office of Politecnico di Milano (authorization 16/2020), and all the subjects gave informed consent according to the declaration of Helsinki.

### Performance metrics and statistical analysis

To evaluate the system for autonomous endoscope navigation, we considered both the performance of the user related to the execution of the experiment and their posterior feedback about the usability of the system. The metrics we investigated were the (i) total execution time (t_tot) spent by each user in performing the experimental version of the orthotopic neobladder reconstruction; (ii) the time out of the field of view (t_out_FoV), which means the cumulative time with at least one of two tools out of the endoscope field of view (i.e., not visible in the image) while carrying out the procedure (Jarc and Curet, 2017); (iii) the results of the System Usability Scale (SUS) survey (Brooke 1996), a validated set of questions whose answers are used to compute a score related to the usability of the system.

The total execution time and the time out of the field of view in both the endoscope modalities (classic endoscope manipulation vs. autonomous endoscope navigation) were analyzed by using the Wilcoxon Rank Sum statistical test to assess significant differences.

## Results

This section reports the results from the 10 users (3 surgical residents and 7 specialists in urology) who completed the study. Their details are shown in the upper part of Figure 5. Users are ordered according to their total number of robotic surgical procedures (as first operator) performed in the last year.

### Total execution Time

The total execution time (t_tot) for the neobladder reconstruction is shown in the second block of Figure 5. As predicted, the total execution time (both in the manual and autonomous endoscope control modalities) is characterized by a decreasing trend as a function of the experience of the user (i.e., the total number of robotic surgical procedures performed in the last year). The average total execution time was assessed when the users were assisted by the autonomous endoscope navigation with respect to classic endoscope manipulation (*p* = 0.85, average t_tot_M = 34 ± 13min vs. average t_tot_A = 33 ± 12min).

If we group the users into three clusters (by considering the residents, and splitting the specialists into fellows and attendings), we can notice how the major benefit in terms of total execution time was gained by the less experienced classes, i.e., the residents and the fellows. In fact, the average time improvement from the manual to the autonomous modality was equal to 1 min and 49 s for the residents and 1 min and 41 s for the fellows. On the other side, the attendings underwent an average time loss of 2 min and 44 s when using the autonomous endoscope.

### Time out of the field of view

The time out of the field of view (t_out_FoV) for the neobladder reconstruction is shown in the third block of Figure 5. This metric can provide an indication of the quality of our autonomous system (i.e., whether it was capable to meet its design principle and to minimize the t_out_FoV). The reduction in time spent by the user with at least one tool out of the field of view during the autonomous navigation with respect to the manual one was statistically significant (*p* = 0.022).

By considering the specialization groups introduced in the previous section (residents, fellows, and attendings), the average influence of the autonomous endoscope control on the time out of the field of view was more remarkable for the residents (average difference from manual to autonomous in t_out_FoV equal to 9 min and 16 s for the residents, 3 min and 37 s for the fellows, and 5 min and 10 s for the attendings).

### System Usability Scale

The System Usability Scale (SUS) outcomes of our system for autonomous endoscope navigation are shown in the bottom block of Figure 5. An average of 74% in the system usability was obtained for the autonomous navigation system across all the subjects. The good usability threshold is commonly defined as >68% (Brooke 1996).

A well-defined trend can be observed if considering the specialization groups (residents, fellows, and attendings). The average SUS score was 91% if considering the residents, 73% if considering the fellows, and 57% if considering the attendings.

## Discussion

In this study, we investigated the usability and performance of an autonomous endoscope navigation system, the SCAN, during *ex vivo* orthotopic neobladder reconstruction.

### Overview of major findings

The average time out of the field of view was reduced while undergoing autonomous endoscope motion. This is in line with the functioning principle of the SCAN as well as with the results obtained in our previous works (Mariani et al., 2020; Da Col et al., 2020), showing a good implementation of the autonomous system in the research version of the da Vinci Surgical System®.

We also found a reduction in the average total execution time for residents and fellows undergoing autonomous endoscope navigation, yet such a reduction was not statistically significant at the group level. The high variability in the overall total execution time (both manual and autonomous navigation), as well as the small sample size of the population under investigation, could be the major explanation of this lack of statistical significance.

This study was designed to primarily investigate the feasibility and the usability of our system in a surgically relevant scenario. The most relevant outcome in this regard is represented by the average score from the system usability scale that overcame the usability threshold (74%) (Brooke 1996).

Some remarkable trends can be derived from the clusterization of users according to their level of expertise (quantified as the total number of robotic procedures as first operator in the previous year). The surgical residents were the ones who benefited the most from the autonomous navigation system of the endoscope and the ones who gave the highest rate to its usability. Such a negative correlation between the operating room experience and our objective and subjective metrics could be related to the existence of a know-how bias. In other words, the habit of using manual navigation of the endoscope in everyday surgical practice plays a relevant role in the acceptance and the effects of a novel control like the autonomous endoscope navigation. To sum up, the results led to a twofold consideration: on the one side, the low usability (SUS) score for the more experienced surgeons can be linked to their already well-accustomed and rooted proficiency with the manual endoscope control, which might make the SCAN system feel as a disruption of the traditional robot-assisted surgery workflow. On the other side, young or early career surgeons, since they are the ones who performed less interventions and received less training in traditional robotic surgery, might be the ones not only adapting more smoothly to the new technology, but also benefiting the most from it.

Nevertheless, SCAN represents a proof of concept for the integration of autonomous endoscope navigation in robotic surgery, and the very final goal is not substituting the current navigation modality, but rather proposing an additional aid to the surgeon. This way, autonomous robotics can enter the operating room as a collaborative tool that the surgeon gets the assistance of, just if desired, and always maintaining full supervision over it.

### Limitations and future developments

Even though this work presented a first feasibility study on a urological scenario, the SCAN technology is still far from the standards required in an operating room. Several limitations of this study and possible future implementations can be highlighted.

The autonomous navigation algorithm tested shown a potential for future clinical applications, yet we tested, in a surgically relevant scenario, only two navigation modalities and some basic forms of user parameter customization. A more tailored yet flexible system, based not only on the initial preferences but also on the intraoperative selections of the surgeon, combined with an improved user interface, could bring autonomous surgical assistance to the next level.

The surgical procedure was a simplified version of the clinical orthotopic neobladder reconstruction where many steps have been removed in order to optimize the waste of resources and the quick regeneration of the setup in case of subsequent data acquisitions. Future improvement of the setup will involve integrating the procedural missing steps to achieve full consistency with the real procedure.

The overall study, exploratory in its nature, aimed at further understanding the potentialities of a semi-autonomous control system and how differently skilled surgeons reacted to a major change in the robotic-assisted surgery teleoperation workflow. We understood that different experience levels have a strong and multifactorial influence on the outcomes of our investigation. Consequently, this highly variable skill level adding up to the relatively small user sample size might have reduced the statistical power of our tests. A larger sample size in parallel to a specific focus on a skill target could be the key ingredients for the success of future investigations. Furthermore, neither a comprehensive quantitative performance assessment nor a full validation of the proposed experimental setup was in the objectives of this work.

A more structured data acquisition protocol and further evaluation of the reconstructed neobladders (by one or more experts) can yield more salient insights on the advantages (or disadvantages) introduced by semi-autonomous endoscope navigation systems in the robotic surgery workflow.

## Conclusion

This work deals with the optimization of the surgical workflow by means of robotic assistance. Specifically, we introduce the SCAN, a system for autonomous endoscope navigation, and we report its testing in a urological scenario (ex-vivo orthotopic neobladder reconstruction). The feedback from 10 urologists (with heterogeneous experience) showed promising results in terms of usability of the system. Performance improved for resident and fellow (but not for attending) surgeons resulting in an average task completion time reduction when undergoing autonomous endoscope control.

To conclude, our system for autonomous endoscope navigation can improve the standard framework of manual camera control by optimizing the surgical workflow. Additionally, our framework has the potential to leave freedom to the surgeons about the activation of autonomous navigation in alternative to the classic manual control, leaving them in power over the control modality and giving them the possibility to customize the autonomous navigation as needed. Moving from interviews with clinicians and further usability studies, future work can investigate the addition of novel modalities of autonomous control that can be selected according to the surgical task.

## Data Availability

The raw data supporting the conclusions of this article will be made available by the authors, without undue reservation.
